# Serum Creatine Levels as a Predictive Factor for Postoperative Cerebrovascular Events in Patients With Moyamoya Disease

**DOI:** 10.1002/brb3.70331

**Published:** 2025-02-11

**Authors:** Siqi Mou, Zhikang Zhao, Chenglong Liu, Junsheng Li, Qiheng He, Wei Liu, Bojian Zhang, Zhiyao Zheng, Wei Sun, Xiangjun Shi, Qian Zhang, Rong Wang, Yan Zhang, Peicong Ge, Dong Zhang

**Affiliations:** ^1^ Department of Neurosurgery Beijing Tiantan Hospital, Capital Medical University Beijing China; ^2^ Medical School University of Chinese Academy of Sciences Beijing China; ^3^ China National Clinical Research Center for Neurological Diseases Beijing China; ^4^ Department of Neurosurgery, Beijing Hospital National Center of Gerontology Beijing China; ^5^ Institute of Geriatric Medicine, Chinese Academy of Medical Sciences Beijing China

**Keywords:** biomarker, creatine, moyamoya disease, outcome

## Abstract

**Background:**

Creatine is essential for energy storage and transfer within and outside cells. However, its relationship with cerebrovascular disease has not been fully explored. This study examined the association between serum creatine levels and postoperative cerebrovascular events, including transient ischemic attack (TIA), ischemic stroke, and hemorrhagic stroke, in patients with moyamoya disease (MMD).

**Methods:**

Serum creatine and disodium creatine phosphate levels were quantified in 352 patients with MMD using liquid chromatography–tandem mass spectrometry. Kaplan–Meier (KM) curves were used to analyze the impact of serum creatine levels on cerebrovascular event risk, whereas univariate and multivariate Cox regression analyses were used to identify predictors of postoperative outcomes. A prognostic nomogram was developed to predict stroke‐free survival at 12, 24, and 36 months postoperatively.

**Results:**

In patients with MMD, serum creatine showed a negative correlation with creatinine (*r* = −0.22; *p* < 0.001) and homocysteine (*r* = −0.10; *p* < 0.05) but not with disodium creatine phosphate (*r* = −0.08; *p* = 0.15). When patients were divided into high and low groups based on the median serum creatine concentration, KM curve analysis revealed that patients in the high concentration group had a lower relative risk of cerebrovascular events than those in the low concentration group (hazard ratio: 0.55; 95% confidence interval, 0.33–0.94; *p* = 0.026). Furthermore, when patients were categorized into three levels based on creatine concentration, the overall KM curve analysis showed a significant difference (*p* = 0.038), such that the highest creatine concentration group (third tertile) showed a significantly reduced risk compared with the lowest concentration group (first tertile; *p* = 0.04).

**Conclusion:**

Lower preoperative serum creatine levels were associated with a higher risk of postoperative cerebrovascular events in patients with MMD. Therefore, creatine supplementation may be an effective means of preventing adverse outcomes in patients with MMD.

## Graphical abstract

This study of 352 moyamoya disease patients revealed that lower preoperative serum creatine levels are significantly associated with an increased risk of postoperative cerebrovascular events. Furthermore, a prognostic model was developed to predict stroke‐free survival over 12–36 months. These findings raise the possibility that creatine supplementation could be beneficial in reducing postoperative cerebrovascular risk, warranting further investigation.

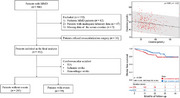

## 1 Background

Moyamoya disease (MMD) is a chronic cerebrovascular disorder with unclear etiology that is characterized by progressive narrowing or occlusion of the terminal portion of the internal carotid arteries and their branches, accompanied by compensatory dilatation of the basal collateral arteries (Suzuki and Takaku 1969; Scott and Smith 2009). Although its incidence rate is relatively low, it is the predominant cause of stroke in children and adolescents in East Asian regions (Kim 2016). Currently, extracranial–intracranial bypass surgery is regarded as the principal therapeutic strategy for patients with MMD, focusing primarily on the restoration or enhancement of cerebral blood flow, symptom mitigation, and diminution of future stroke risk (El Naamani et al. 2024). The prognosis of MMD is individual‐specific and is influenced by multiple factors. Recent studies have demonstrated that adverse postoperative outcomes after vascular reconstruction surgery are correlated with variables including genetic predisposition, inflammatory biomarkers, nutritional condition of the patient, and surgical method (Fang et al. 2024; W. Liu, Sun, et al. 2023; W. Liu, Huang, et al. 2023; Yuan et al. 2024). Therefore, early diagnosis and appropriate treatment are crucial to improve patient outcomes. The exploration of risk factors for negative prognostic events seeks to reduce the occurrence of adverse cerebrovascular events postoperatively.

Creatine is classified with amino acids and their derivatives (Wallimann et al. 2011). It can be obtained via dietary intake or synthesized in the body (Joncquel‐Chevalier Curt et al. 2015). Under the catalysis of creatine kinase, creatine combines with adenosine triphosphate (ATP) to generate phosphocreatine, which is crucial for energy storage and intracellular energy transfer (van de Velde et al. 2023). Through nonenzymatic dehydration and cyclization, creatine and phosphocreatine are converted to creatinine, with approximately 1.7% of both substances being transformed into creatinine daily (Wyss and Schulze 2002). This end product is excreted from the body through free diffusion into the urine. Disruptions in creatine metabolism have been documented in conditions such as creatine deficiency syndrome and mitochondrial disorders (Shayota 2024; Jomura et al. 2024). Brain creatine deficiency is associated with neurological dysfunction and structural changes in mitochondria, potentially leading to encephalomyopathies and mitochondrial myopathies (Shaham et al. 2010). Emerging research posits that creatine is a potential novel neurotransmitter, with diminished brain creatine levels possibly impairing central nervous system function (Bian et al. 2023). Previous studies have shown that creatine supplementation improves cognitive function in elderly participants (McMorris et al. 2007). Creatine levels in the peripheral circulation partially mirror the body's total creatine content. Mitochondrial dysfunction has been observed in circulating endothelial colony‐forming cells of patients with MMD, and homozygous variants in *TOMM7* (translocase of the outer mitochondrial membrane 7) can lead to microcephalic osteodysplastic dwarfism accompanied by MMD (Choi et al. 2018; Li et al. 2024). Therefore, in the context of mitochondrial dysfunction in patients with MMD, the relationship between changes in serum creatine levels and prognosis remains unknown and warrants further investigation.

This study aimed to identify the risk factors for adverse cerebrovascular events in patients with MMD during the long‐term follow‐up period after surgery and to examine the association between serum creatine concentrations and adverse patient outcomes, further elucidating the potential pathophysiological mechanisms involved.

## 2 Methods

### 2.1 Study Participants

**FIGURE 1:**
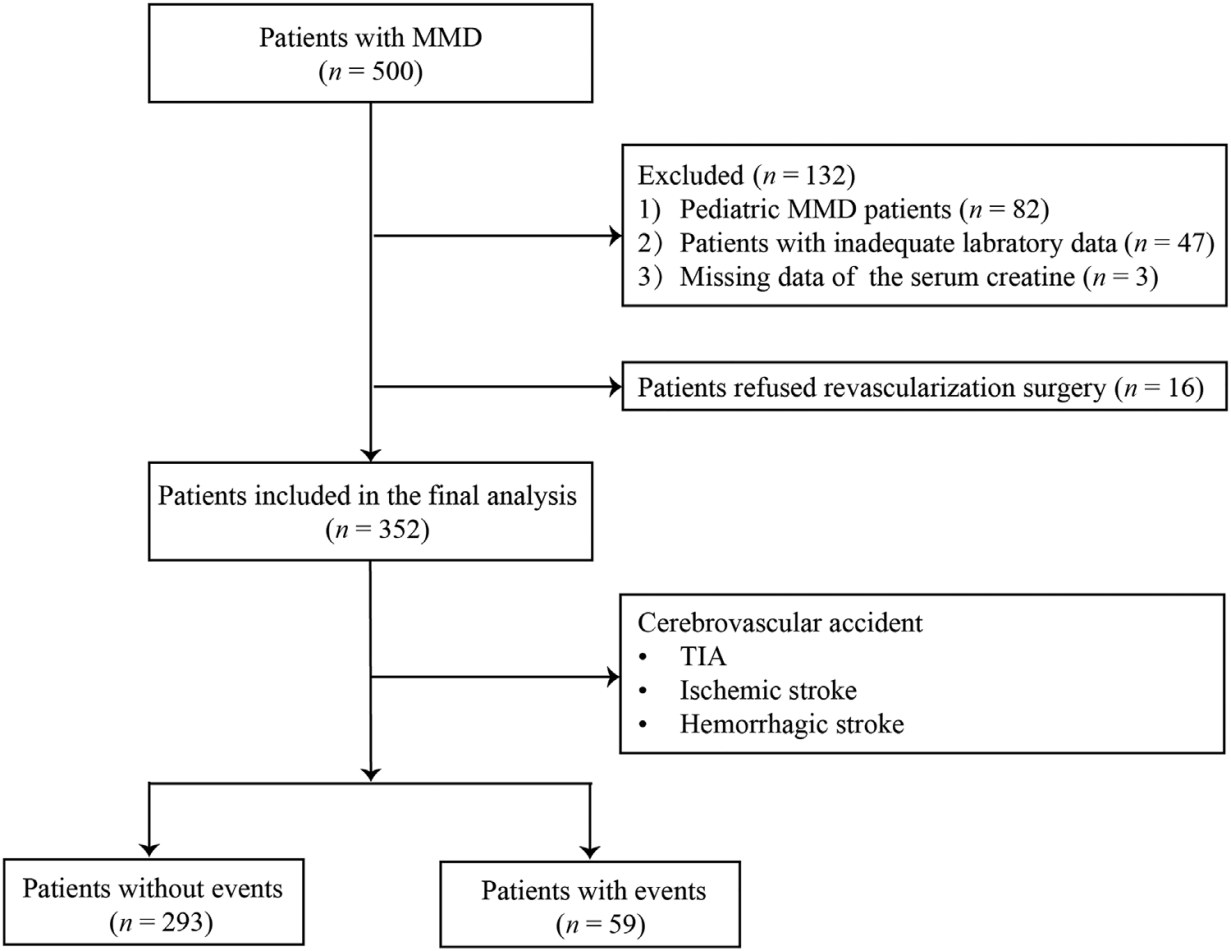
Flow diagram of patient selection. MMD, moyamoya disease; TIA, transient ischemic attack.

In this prospective study, we consecutively enrolled 500 patients with MMD between September 2020 and December 2021. All patients were diagnosed with MMD based on the 2012 Japanese diagnostic criteria and confirmed by digital subtraction angiography (DSA) (Research Committee on the Pathology and Treatment of Spontaneous Occlusion of the Circle of Willis and Health Labour Sciences Research Grant for Research on Measures for Infractable Diseases 2012). The study excluded 82 pediatric patients, 47 patients due to insufficient laboratory data, 3 patients with missing serum creatine levels, and 16 patients who did not undergo neurosurgical cerebral revascularization. Ultimately, 352 patients were included in the study, of whom 293 did not experience cerebrovascular events during follow‐up and 59 who did (Figure 1). This research falls within the category of multiomics of MMD (Ge et al. 2024). All research protocols were conducted in accordance with the Declaration of Helsinki and were approved by the Ethics Committee of Beijing Tiantan Hospital, Capital Medical University. Written informed consent was obtained from all participants or their legal guardians.

### 2.2 Data Collection

The clinical characteristics of the patients with MMD collected at the time of admission included demographic data (age and sex), baseline data (heart rate, blood pressure, and body mass index [BMI]), personal medical history (hypertension, diabetes, hyperlipidemia, smoking, and alcohol consumption), and initial clinical presentations (ischemic and hemorrhagic). BMI was calculated as weight (kg) divided by height squared (m^2^). Neurological status was assessed using the modified Rankin Scale (mRS) score at admission, with scores categorized into two groups (0–2 and 3–5).

### 2.3 Blood Sample Analysis

After a minimum fasting period of 12 h, peripheral venous blood samples were collected from patients with MMD at 8:00 a.m. A portion of peripheral blood was used for routine and biochemical laboratory tests, as detailed in Table 1. The remaining peripheral blood was centrifuged at 3000 × *g* for 10 min, and the serum samples were stored at −80°C for subsequent sequencing analysis. Consistent with previous studies, the concentrations of creatine and disodium creatine phosphate in the serum of each patient were determined using liquid chromatography‐tandem mass spectrometry (C. Liu, Ge, et al. 2023).

**TABLE 1:** Baseline characteristics and laboratory examinations of patients followed up after surgery.

	Follow‐up		
Characteristics	Patients without events (*n* = 293)	Patients with events (*n* = 59)	p value
General conditions			
Age (years; mean ± SD)	41.99 ± 10.53	42.34 ± 11.29	0.820
Female, *n* (%)	167 (57.0)	37 (62.7)	0.417
Heart rate (bpm; mean ± SD)	78.70 ± 6.61	79.05 ± 5.74	0.701
SBP (mmHg; mean ± SD)	132.05 ± 13.18	136.07 ± 14.84	0.038^*^
DBP (mmHg; mean ± SD)	81.82 ± 9.35	83.58 ± 10.45	0.197
BMI (kg/m^2^; mean ± SD)	25.59 ± 4.74	24.86 ± 3.43	0.265
History of risk factors, *n* (%)			
Smoking	57 (19.5)	13 (22.0)	0.651
Drinking	34 (11.6)	7 (11.9)	0.955
Diabetes mellitus	46 (15.7)	11 (18.6)	0.575
Hypertension	103 (35.2)	26 (44.1)	0.195
Hyperlipidemia	36 (12.3)	13 (22.0)	0.048^*^
Laboratory tests, median (IQR)			
Hematological parameters			
WBC count (10^9^/L)	6.81 (2.49)	6.90 (2.57)	0.694
Lymphocyte count (10^9^/L)	1.91 (0.86)	1.89 (0.95)	0.243
Neutrophil count (10^9^/L)	4.20 (1.81)	4.32 (2.14)	0.374
Platelet count (10^9^/L)	249.00 (77.00)	246.00 (79.00)	0.408
RBC count (10^12^/L)	4.64 (0.63)	4.47 (0.91)	0.060
Coagulation function			
FDP (µg/mL)	1.30 (1.14)	1.60 (1.58)	0.185
D‐dimer (µg/mL)	0.55 (0.46)	0.50 (0.49)	0.570
PT (s)	11.40 (1.00)	11.40 (1.30)	0.920
PT (INR)	1.01 (0.10)	1.01 (0.12)	0.611
APTT (s)	30.60 (3.70)	30.60 (4.60)	0.636
TT (s)	14.30 (1.30)	14.30 (1.50)	0.609
FIB (g/L)	2.90 (0.75)	2.83 (0.80)	0.879
Blood glucose and lipid			
Glucose (mmol/L)	5.13 (1.06)	4.96 (1.20)	0.133
TC (mmol/L)	4.23 (1.31)	4.33 (1.56)	0.757
TG (mmol/L)	1.22 (0.84)	1.15 (0.83)	0.292
HDL‐C (mmol/L)	1.31 (0.37)	1.28 (0.40)	0.495
LDL‐C (mmol/L)	2.40 (1.14)	2.40 (1.27)	0.898
apoA (g/L)	1.29 (0.28)	1.36 (0.37)	0.155
apoB (g/L)	0.82 (0.28)	0.82 (0.30)	0.448
Liver and kidney function			
Albumin (g/L)	45.50 (3.85)	45.50 (4.50)	0.863
Alkaline phosphatase (U/L)	69.50 (25.95)	67.00 (30.20)	0.811
GGT (mmol/L)	23.60 (19.45)	22.00 (14.00)	0.495
Urea (mmol/L)	4.60 (1.80)	4.40 (1.70)	0.731
Total CO_2_ (mmol/L)	25.00 (3.60)	24.00 (3.80)	0.010^*^
eGFR (mL/min)	123.45 (20.25)	125.60 (20.09)	0.645
Hcy (µmol/L)	12.29 (5.85)	11.23 (5.93)	0.474
SII	528.37 (372.28)	638.67 (529.14)	0.066
Creatinine (µmol/L)	55.50 (19.65)	54.20 (22.60)	0.624
Disodium creatine phosphate (µmol/L)	138.21 (134.36)	154.60 (126.77)	0.344
Creatine (µmol/L)	69.32 (35.94)	62.28 (28.79)	0.161
Creatine (median split)			0.032^*^
Low	139 (47.4)	37 (62.7)	
High	154 (52.6)	22 (37.3)	
Creatine tertile			0.055
Tertile 1	92 (31.4)	25 (42.4)	
Tertile 2	98 (33.4)	20 (33.9)	
Tertile 3	103 (35.2)	14 (23.7)	
Primary symptom, *n* (%)			0.549
Hemorrhage	83 (28.3)	19 (32.2)	
Ischemia	210 (71.7)	40 (67.8)	
Suzuki stage, *n* (%)			
≤ 3	179 (61.1)	33 (55.9)	0.460
> 3	114 (38.9)	26 (44.1)	
PCA involvement, *n* (%)	38 (13.0)	9 (15.3)	0.638
Admission mRS, *n* (%)			0.373
0–2	262 (89.4)	55 (93.2)	
3–5	31 (10.6)	4 (6.8)	
Surgical option, *n* (%)			0.559
Indirect bypass	171 (58.4)	32 (54.2)	
Nonindirect bypass	122 (41.6)	27 (45.8)	

Abbreviations: apoA, apolipoprotein A; apoB, apolipoprotein B; APTT, activated partial thromboplastin time; BMI, body mass index; DBP, diastolic blood pressure; eGFR, estimated glomerular filtration rate; FDP, fibrinogen degradation products; FIB, fibrinogen; GGT, γ‐glutamyltransferase; Hcy, homocysteine; HDL‐C, high‐density lipoprotein cholesterol; INR, international normalized ratio; IQR, interquartile range; LDL‐C, low‐density lipoprotein cholesterol; mRS, modified Rankin Scale; PCA, posterior cerebral artery; PT, prothrombin time; RBC, red blood cell; SBP, systolic blood pressure; SD, standard deviation; SII, systemic immune‐inflammation index; TC, total cholesterol; TG, triglyceride; TT, thrombin time; WBC, white blood cell.

* *p *< 0.05, significant difference.

### 2.4 Imaging Evaluation and Surgical Procedures

Two neurosurgeons independently evaluated the Suzuki stage (based on the more severely affected side) and assessed the involvement of the posterior cerebral artery using DSA (Luo et al. 2023). In case of disagreement, the two experts discussed the findings to reach a consensus.

The following three surgical methods were employed: direct, indirect, and combined revascularization procedures, as detailed in our previous studies (Deng et al. 2018). Briefly, direct bypass involves the anastomosis of a cortical branch of the middle cerebral artery (MCA) with the superficial temporal artery (STA). In indirect bypass, branches of the STA are dissected and placed on the cerebral cortical surface. Combined bypass involves the simultaneous performance of both direct and indirect bypasses in the same hemisphere. The direct and combined bypass approaches are preferred at Beijing Tiantan Hospital. Indirect bypass surgery was performed if the STA or MCA was too fragile for anastomosis. The primary consideration for revascularization surgery was the patient's clinical symptoms, particularly in symptomatic hemispheres. For asymptomatic patients, computed tomography (CT) perfusion imaging was used to guide treatment decisions, particularly for hemispheres with insufficient perfusion.

### 2.5 Clinical Outcomes

Long‐term outcomes were assessed using a combination of face‐to‐face visits and telephone interviews conducted 11–47 months after discharge. Specifically, all patients were scheduled for in‐person evaluations at 12, 24, and 36 months postoperatively. Structured telephone interviews were conducted at 6‐month intervals to accommodate those unable to attend in‐person visits. The follow‐up methods were tailored based on patient availability and geographical location, ensuring that each patient underwent at least one face‐to‐face evaluation during the follow‐up period.

Events recorded during the follow‐up period included transient ischemic attack (TIA), ischemic stroke, and hemorrhagic stroke. The timing of TIA events was recorded based on their first occurrence postoperatively. Both follow‐up methods employed standardized assessment protocols utilizing the mRS to consistently evaluate neurological status. mRS scores of 0‐2 indicated a favorable neurological state and scores of 3‐5 indicated an adverse neurological state. All evaluators underwent specialized training to ensure the reliability of the data collection. Neuroradiological assessments were performed exclusively during face‐to‐face visits, with high‐risk patients identified via telephone interviews receiving prioritized in‐person evaluations. Additionally, all evaluators were blinded to patients' serum creatine levels and other biochemical data to maintain objectivity in outcome assessments.

### 2.6 Statistical Analysis

Statistical analyses were conducted using SPSS (version 26.0) and R (version 4.1.3; https://www.r‐project.org). A complete case analysis was conducted on the participants included in the study. Categorical variables are presented as frequencies, whereas continuous variables are described using means and standard deviations (SDs) or medians with interquartile ranges (IQRs). Continuous data between the two groups were compared using the *t*‐test or Mann–Whitney *U* test. Multiple groups were compared using one‐way analysis of variance (ANOVA). Categorical variables were compared using Pearson's chi‐square test and the Cochran–Armitage trend test.

The relationships between serum creatine concentrations and creatinine, homocysteine (Hcy), and disodium creatine phosphate levels were evaluated using Spearman's correlation test. Kaplan–Meier (KM) survival curves were used to analyze the different serum creatine concentration groups, followed by the log‐rank test. Univariate Cox regression analysis was performed to identify prognostic predictors of postoperative events in patients with MMD. Different serum creatine concentrations were investigated as independent risk factors using a multivariate Cox proportional hazards regression model. In univariate Cox regression, variables with a *p* value less than 0.01 were included in the multivariate model using backward regression to minimize the Akaike information criterion (AIC) value, identifying systolic blood pressure (SBP), hyperlipidemia, red blood cell (RBC) count, fibrin degradation products (FDP), total carbon dioxide (CO_2_), systemic immune‐inflammation index (SII), and creatine tertile as model variables. The effect of creatine concentration tertiles in the model was assessed using the time‐dependent area under the curve (time‐AUC) with a bootstrap value of 500. A prognostic nomogram was generated to evaluate the probability of stroke‐free survival at 12, 24, and 36 months postoperatively.

## 3 Results

### 3.1 Study Participants and Baseline Characteristics

This study included 352 patients with MMD, divided into the following two groups: 293 patients who did not experience cerebrovascular events during the postoperative follow‐up period and 59 patients who did. Table 1 compares the participants’ basic characteristics and laboratory examination results. Compared to the group without follow‐up events, the group with events exhibited higher SBP and lower levels of total CO_2_, as well as a greater propensity for hyperlipidemia (*p *< 0.05 for all). Although creatine concentration in the group with events was lower than that in the group without events, the difference was not statistically significant (*p = *0.161). However, when the patients were divided into high and low groups based on the median serum creatine concentration, a higher proportion of patients with low creatine concentrations were observed in the group with events (*p = *0.032). Furthermore, when dividing patients based on tertiles of serum creatine concentration, the group with events had a higher proportion of patients with low creatine concentrations and a lower proportion of patients with high creatine concentrations, and this trend approached statistical significance (*p = *0.055).

**FIGURE 2:**
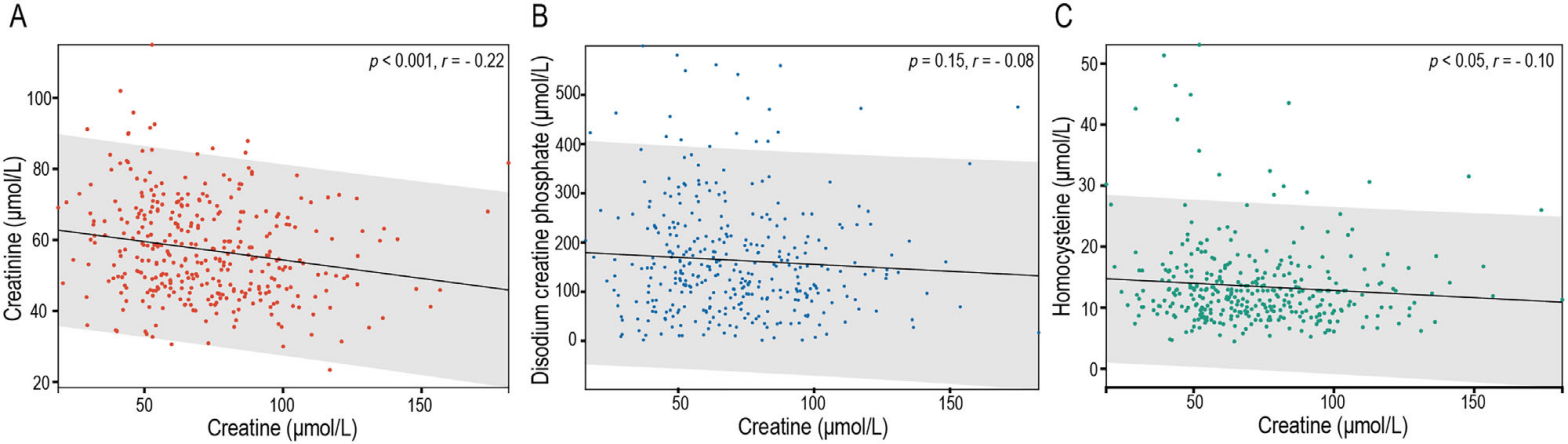
Linear correlation between serum creatine and creatinine, disodium creatine phosphate, and homocysteine: (A) creatinine, (B) disodium creatine phosphate, and (C) homocysteine. *p* values were calculated using Spearman's correlation analysis, and values less than 0.05 were considered statistically significant.

After dividing all patients into tertiles based on serum creatine levels, patients with higher creatine levels were predominantly female and exhibited lower creatinine and Hcy levels (*p *< 0.05 for all; Table 2). Additionally, serum creatine concentration, as a continuous variable, showed a negative linear relationship with creatinine and Hcy levels, whereas there was no significant linear relationship with the concentration of disodium creatine phosphate (Figure 2).

**TABLE 2:** Baseline characteristics of participants according to the tertiles of the serum creatine concentrations.

Characteristics	Tertiles of changes in serum creatine			*p* for trend
	T1 (< 56.61)	T2 (56.61–< 80.91)	T3 (≥ 80.91)	
No. of participants	117	118	117	
Creatine (µmol/L)	48.81 (11.59)	68.49 (11.98)	97.69 (22.44)	< 0.001^*^
Creatinine (µmol/L)	60.50 (21.30)	54.25 (17.85)	52.10 (18.40)	< 0.001^*^
Disodium creatine phosphate (µmol/L)	148.75 (152.09)	143.19 (118.63)	129.15 (120.64)	0.645
General conditions				
Age (years; mean ± SD)	41.97 ± 10.41	41.21 ± 11.87	42.98 ± 9.52	0.466
Female, *n* (%)	51 (43.6)	69 (58.5)	84 (71.8)	< 0.001^*^
Heart rate (bpm; mean ± SD)	78.96 ± 6.83	79.21 ± 6.14	78.09 ± 6.42	0.308
SBP (mmHg; mean ± SD)	132.61 ± 13.73	132.75 ± 14.19	132.82 ± 12.76	0.904
DBP (mmHg; mean ± SD)	83.24 ± 9.15	80.86 ± 10.64	82.24 ± 8.66	0.423
BMI (kg/m^2^; mean ± SD)	25.36 ± 4.13	25.58 ± 5.04	25.44 ± 4.46	0.878
History of risk factors, *n* (%)				
Smoking	28 (23.9)	25 (21.2)	17 (14.5)	0.072
Drinking	13 (11.1)	13 (11.0)	15 (12.8)	0.684
Diabetes mellitus	17 (14.5)	16 (13.6)	24 (20.5)	0.215
Hypertension	47 (40.2)	40 (33.9)	42 (35.9)	0.498
Hyperlipidemia	21 (17.9)	15 (10.2)	16 (13.7)	0.346
Laboratory tests, median (IQR)				
Hematological parameters				
WBC count (10^9^/L)	6.88 (2.33)	6.90 (2.94)	6.77 (2.38)	0.274
Lymphocyte count (10^9^/L)	2.00 (0.81)	1.90 (0.90)	1.86 (0.92)	0.085
Neutrophil count (10^9^/L)	4.15 (1.72)	4.32 (1.97)	4.11 (2.02)	0.719
Platelet count (10^9^/L)	243.00 (72.50)	248.00 (75.75)	251.00 (85.00)	0.632
RBC count (10^12^/L)	4.74 (0.74)	4.61 (0.68)	4.57 (0.68)	0.147
Coagulation function				
FDP (µg/mL)	1.23 (1.08)	1.41 (1.19)	1.40 (1.28)	0.181
D‐dimer (µg/mL)	0.56 (0.48)	0.54 (0.55)	0.54 (0.44)	0.289
PT (s)	11.40 (1.10)	11.40 (1.00)	11.20 (1.10)	0.553
PT (INR)	1.02 (0.10)	1.02 (0.11)	1.01 (0.11)	0.490
APTT (s)	30.60 (3.00)	30.50 (3.93)	30.70 (4.20)	0.913
TT (s)	14.40 (1.45)	14.30 (1.13)	14.30 (1.50)	0.122
FIB (g/L)	2.83 (0.78)	2.98 (0.74)	2.95 (0.71)	0.347
Blood glucose and lipid				
Glucose (mmol/L)	5.06 (1.15)	5.06 (0.82)	5.15 (1.15)	0.631
TC (mmol/L)	4.12 (1.36)	4.23 (1.36)	4.23 (1.19)	0.456
TG (mmol/L)	1.17 (1.09)	1.24 (1.04)	1.20 (0.63)	0.335
HDL‐C (mmol/L)	1.26 (0.36)	1.34 (0.41)	1.33 (0.35)	0.231
LDL‐C (mmol/L)	2.31 (1.04)	2.40 (1.16)	2.40 (1.22)	0.625
apoA (g/L)	1.25 (0.31)	1.31 (0.27)	1.30 (0.36)	0.122
apoB (g/L)	0.82 (0.30)	0.82 (0.28)	0.82 (0.30)	0.983
Liver and kidney function				
Albumin (g/L)	46.00 (3.90)	45.60 (3.90)	45.00 (3.85)	0.052
Alkaline phosphatase (U/L)	70.00 (26.95)	68.20 (28.80)	68.40 (27.20)	0.515
GGT (mmol/L)	23.40 (18.60)	25.60 (22.33)	21.10 (15.40)	0.436
Urea (mmol/L)	4.60 (1.90)	4.35 (1.60)	4.70 (1.95)	0.816
Total CO_2_ (mmol/L)	25.00 (3.60)	24.55 (2.85)	24.50 (3.60)	0.079
eGFR (mL/min)	122.37 (21.27)	125.31 (20.79)	123.88 (16.45)	0.290
Hcy (µmol/L)	12.60 (6.43)	11.95 (5.06)	11.90 (5.68)	0.043^*^
SII	540.00 (348.37)	550.58 (444.99)	512.53 (358.41)	0.418
Primary symptom, *n* (%)				0.666
Hemorrhage	33 (28.2)	39 (33.1)	30 (25.6)	
Ischemia	84 (71.8)	79 (66.9)	87 (74.4)	
Suzuki stage, *n* (%)				0.790
≤ 3	69 (59.0)	72 (61.0)	71 (60.7)	
> 3	48 (41.0)	46 (39.0)	46 (39.3)	
PCA involvement, *n* (%)	16 (13.7)	20 (16.9)	11 (9.4)	0.337
Admission mRS, *n* (%)				0.383
0–2	105 (89.7)	111 (94.1)	101 (86.3)	
3–5	12 (10.3)	7 (5.9)	16 (13.7)	
Surgical option, *n* (%)				1.000
Indirect bypass	69 (59.0)	65 (55.1)	67 (59.0)	
Nonindirect bypass	48 (41.0)	53 (44.9)	48 (41.0)	

Abbreviations: apoA, apolipoprotein A; apoB, apolipoprotein B; APTT, activated partial thromboplastin time; BMI, body mass index; DBP, diastolic blood pressure; eGFR, estimated glomerular filtration rate; FDP, fibrinogen degradation products; FIB, fibrinogen; GGT, γ‐glutamyltransferase; Hcy, homocysteine; HDL‐C, high‐density lipoprotein cholesterol; INR, international normalized ratio; IQR, interquartile range; LDL‐C, low‐density lipoprotein cholesterol; mRS, modified Rankin Scale; PCA, posterior cerebral artery; PT, prothrombin time; RBC, red blood cell; SBP, systolic blood pressure; SD, standard deviation; SII, systemic immune‐inflammation index; TC, total cholesterol; TG, triglyceride; TT, thrombin time; WBC, white blood cell.

* *p *< 0.05, significant difference.

### 3.2 The Correlation Between Creatine Levels and Long‐Term Adverse Cerebrovascular Events Postoperatively

In this study, the patients were categorized into three groups based on their serum creatine concentration, with each group having an average follow‐up duration of approximately 20 months. The results indicated a trend toward a decrease in the incidence of TIA and cerebrovascular events during the postoperative follow‐up period as creatine levels increased, although this trend was not statistically significant (*p = *0.070 and *p = *0.055, respectively). Additionally, there was no statistically significant difference in long‐term mRS scores between the different creatine concentration groups (*p = *0.883; Table 3).

**TABLE 3:** Long‐term outcomes of patients with MMD according to tertiles of changes in serum creatine.

Characteristics	Tertiles of changes in serum creatine			*p* for trend
	T1 (< 56.61)	T2 (56.61–< 80.91)	T3 (≥ 80.91)	
No. of participants	117	118	117	
Follow‐up (months; mean ± SD)	19.99 ± 6.82	19.89 ± 6.39	20.78 ± 7.33	0.381
Follow‐up events, *n* (%)				
TIA	19 (16.2)	13 (11.0)	10 (8.5)	0.070
Ischemic stroke	4 (3.4)	4 (3.4)	4 (3.4)	1.000
Hemorrhagic stroke	1 (0.9)	3 (2.5)	1 (0.9)	1.000
Cerebrovascular accident	25 (21.4)	20 (16.9)	14 (12.0)	0.055
Long‐term mRS, *n* (%)				0.883
0–2	110 (94.0)	113 (95.8)	111 (94.9)	
3–5	7 (6.0)	5 (4.2)	6 (5.1)	

Abbreviations: mRS, modified Rankin Scale; SD, standard deviation; TIA, transient ischemic attack.

**FIGURE 3:**
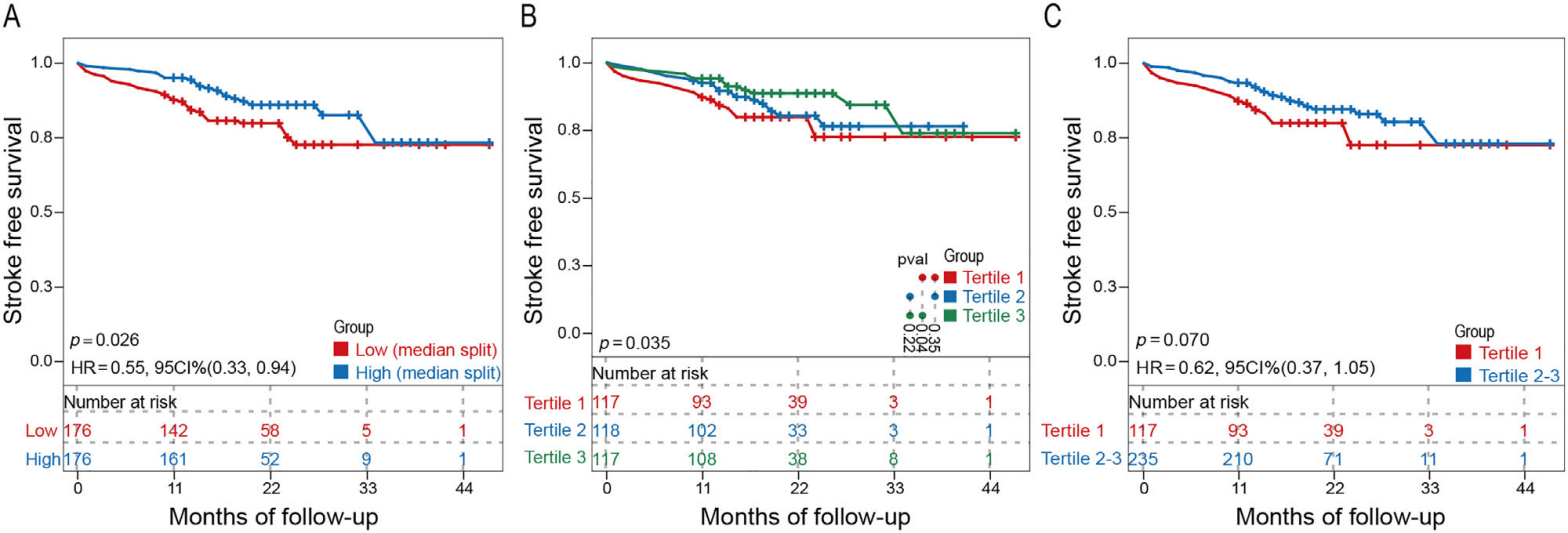
Kaplan–Meier cumulative hazard curve for stroke occurrence across the grouping of different creatine concentrations: (A) median split; (B) Tertiles 1, 2, and 3; and (C) Tertile 1 and Tertiles 2 and 3. *p* < 0.05, significant difference.

When patients were divided into high and low concentration groups based on the median serum creatine concentration, the KM curve analysis demonstrated that patients in the high creatine concentration group had a lower hazard ratio than those in the low concentration group. However, when the analysis was performed with patients divided into tertiles based on creatine concentration, the overall KM curve analysis revealed significant differences (*p = *0.035), with a significantly reduced hazard ratio in the highest creatine concentration group (Tertile 3) compared to the lowest concentration group (Tertile 1; *p = *0.04). Further analysis combining the medium (Tertile 2) and high (Tertile 3) creatine concentration groups showed a reduction in hazard ratio (Figure 3).

**FIGURE 4:**
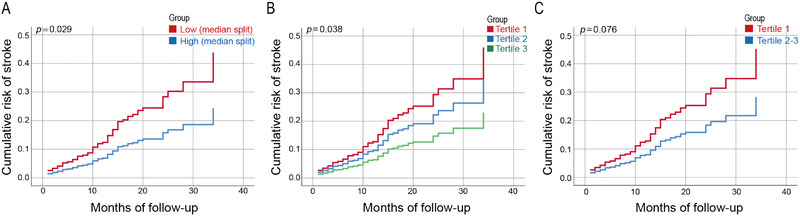
Univariate Cox regression analysis was conducted to examine the cumulative hazard curve for stroke occurrence, categorized by different creatine concentration groups: (A) median split; (B) Tertiles 1, 2, and 3; and (C) Tertile 1 and Tertiles 2 and3. *p *< 0.05, significant difference.

Over time, the cumulative risk of stroke gradually increased in the low creatine concentration group compared with that in the high concentration group. Figure 4 shows the cumulative stroke risk curves across different creatine concentration groups, clearly illustrating the trend of reduced cumulative stroke risk with increased creatine levels.

### 3.3 Clinical Prognostic Prediction Model

We analyzed potential factors associated with cerebrovascular events during the postoperative follow‐up period in adult patients with MMD. Univariate Cox analysis revealed that SBP, hyperlipidemia, RBC count, FDP, D‐dimer, alkaline phosphatase, total CO_2_, SII, creatine (median split), and creatine tertiles were significantly associated with the occurrence of postoperative cerebrovascular events (Table 4).

**TABLE 4:** Univariate Cox regression analysis for cerebrovascular events.

Variables	Univariate analysis	
	HR (95% CI)	*p* value
General conditions		
Age	1.002 (0.978–1.027)	0.858
Sex	0.749 (0.442–1.271)	0.284
Heart rate	1.012 (0.975–1.050)	0.542
SBP	1.017 (1.001–1.034)	0.039^*^
DBP	1.013 (0.986–1.039)	0.354
BMI	0.966 (0.908–1.027)	0.264
History of risk factors		
Smoking	1.115 (0.602–2.065)	0.729
Drinking	0.938 (0.426–2.069)	0.875
Diabetes mellitus	1.094 (0.567–2.210)	0.788
Hypertension	1.268 (0.758–2.122)	0.365
Hyperlipidemia	1.973 (1.063–3.661)	0.031^*^
Hematological parameters		
WBC count	1.077 (0.954–1.214)	0.230
Lymphocyte count	0.757 (0.496–1.156)	0.197
Neutrophil count	1.118 (0.994–1.258)	0.062
Platelet count	1.002 (0.999–1.006)	0.242
RBC count	0.574 (0.345–0.955)	0.033^*^
Coagulation function		
FDP	1.127 (1.061–1.197)	< 0.001^*^
D‐dimer	1.205 (1.067–1.361)	0.003^*^
PT	1.003 (0.743–1.356)	0.982
PT(INR)	0.212 (0.007–6.41)	0.373
APTT	1.056 (0.963–1.157)	0.247
TT	0.925 (0.735–1.165)	0.508
FIB	1.040 (0.703–1.539)	0.843
Blood glucose and lipid		
Glucose	0.840 (0.675–1.046)	0.120
TC	1.063 (0.816–1.386)	0.650
TG	0.856 (0.618–1.185)	0.348
HDL‐C	1.867 (0.783–4.450)	0.159
LDL‐C	1.019 (0.752–1.381)	0.903
apoA	1.926 (0.698–5.316)	0.206
apoB	0.694 (0.220–2.190)	0.534
Liver and kidney function		
Albumin	0.986 (0.905–1.074)	0.739
Alkaline phosphatase	1.003 (1.000–1.007)	0.046^*^
GGT	1.004 (0.999–1.010)	0.106
Urea	1.006 (0.832–1.215)	0.953
Total CO_2_	0.843 (0.751–0.934)	0.001^*^
eGFR	0.997 (0.981–1.013)	0.710
Hcy	0.967 (0.922–1.013)	0.158
SII	1.001 (1.000–1.001)	0.005^*^
Primary symptom		
Ischemia	Reference	—
Hemorrhage	1.216 (0.703–2.102)	0.484
Suzuki stage		
≤ 3	Reference	—
> 3	1.197 (0.715–2.003)	0.494
Surgical option		
Nonindirect bypass	Reference	—
Indirect bypass	0.858 (0.514–1.432)	0.557
Creatinine	0.996 (0.978–1.015)	0.756
Disodium creatine phosphate	1.000 (0.999–1.002)	0.727
Creatine (continuous)	0.993 (0.983–1.003)	0.158
Creatine (median split)	0.555 (0.327–0.942)	0.029^*^
Creatine tertile	0.713 (0.518–0.981)	0.038^*^
Tertile 1	Reference	—
Tertile 2	0.756 (0.420–1.361)	0.351
Tertile 3	0.501 (0.260–0.966)	0.039^*^
Tertile 1	1 (Reference)	—
Tertiles 2 and 3	0.626 (0.373–1.050)	0.076

Abbreviations: apoA, apolipoprotein A; apoB, apolipoprotein B; APTT, activated partial thromboplastin time; BMI, body mass index; CI, confidence interval; DBP, diastolic blood pressure; eGFR, estimated glomerular filtration rate; FDP, fibrinogen degradation products; FIB, fibrinogen; GGT, γ‐glutamyltransferase; Hcy, homocysteine; HDL‐C, high‐density lipoprotein cholesterol; HR, hazard ratio; INR, international normalized ratio; LDL‐C, low‐density lipoprotein cholesterol; PT, prothrombin time; RBC, red blood cell; SBP, systolic blood pressure; SII, systemic immune‐inflammation index; TC, total cholesterol; TG, triglyceride; TT, thrombin time; WBC, white blood cell.

* *p *< 0.05, significant difference.

Based on the univariate analysis, we constructed a clinical prognostic prediction model that included the following variables: SBP, hyperlipidemia, RBC count, FDP, total CO_2_, SII, and creatine tertile (Table 5).

**TABLE 5:** Multivariate Cox regression analysis for cerebrovascular events.

Variables	Multivariate analysis	
	HR (95% CI)	*p* value
SBP	1.019 (1.002–1.037)	0.028^*^
Hyperlipidemia	2.163 (1.140–4.103)	0.018^*^
RBC count	0.671 (0.390–1.153)	0.148
FDP	1.129 (1.054–1.210)	0.001^*^
Total CO_2_	0.871 (0.793–0.957)	0.004^*^
SII	1.001 (1.000–1.001)	0.027^*^
Creatine tertile	0.641 (0.458–0.899)	0.010^*^

Abbreviations: CI, confidence interval; FDP, fibrinogen degradation products; HR, hazard ratio; RBC, red blood cell; SBP, systolic blood pressure; SII, systemic immune‐inflammation index.

* *p *< 0.05, significant difference.

**FIGURE 5:**
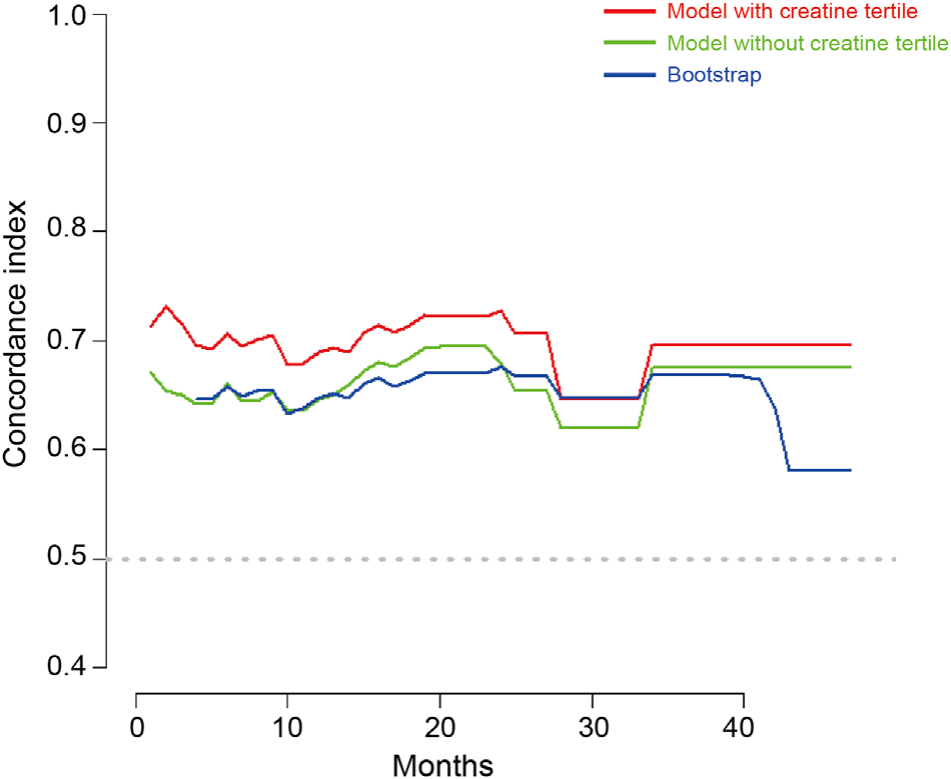
Time‐AUC evaluates the role of the creatine tertile in the model. Bootstrap = 500.

**FIGURE 6:**
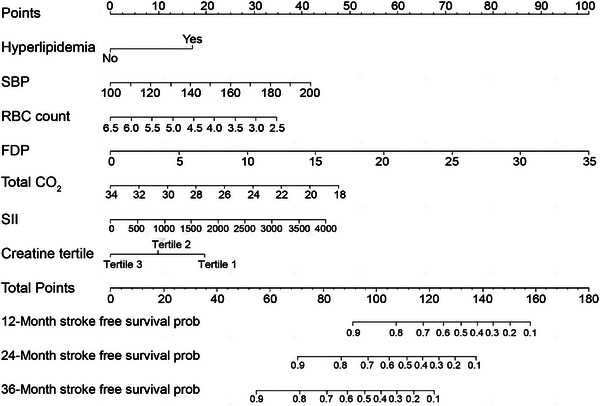
Nomogram of related factors influencing postoperative cerebrovascular events. FDP, fibrinogen degradation products; RBC, red blood cell; SBP, systolic blood pressure; SII, systemic immune‐inflammation index.

Time‐AUC analysis indicated that the creatine tertile in the model increased the AUC value at most time points compared with the models without this variable, as shown in Figure 5. To establish a predictive model for postoperative cerebrovascular events, we constructed a nomogram based on SBP, hyperlipidemia, RBC count, FDP, total CO2, SII, and creatine tertile. This nomogram is shown in Figure 6.

## 4 Discussion

This study aimed to investigate the relationship between serum creatine levels in patients with MMD and the risk of postoperative adverse cerebrovascular events. Our findings suggest that during the follow‐up period, lower serum creatine levels were significantly correlated with an increased risk of postoperative cerebrovascular events. Using preoperative patient data, we developed a clinical prognostic model that effectively identified the variables significantly associated with postoperative cerebrovascular events. The inclusion of creatine levels significantly enhanced the predictive accuracy of the model, offering a potential avenue for guiding treatment decisions and improving the prognosis of patients with MMD based on more precise data. Therefore, creatine supplementation may be an effective strategy to mitigate adverse outcomes in patients with MMD.

Creatine is primarily synthesized in the liver, with the synthesis process consuming methionine and producing Hcy as an intermediate product. Under normal conditions, this metabolic process remains balanced (Van Bavel et al. 2019). Theoretically, if creatine synthesis is reduced, the concentration of Hcy should decrease accordingly. However, in our patient group, low creatine levels were associated with high Hcy levels. This phenomenon could be due to insufficient energy supply due to tissue hypoxia, such that creatine in the peripheral blood is transported into cells in increased amounts to provide energy, thereby disrupting the metabolic balance within the body. Furthermore, studies have indicated that high Hcy levels are associated with an increased risk of developing MMD (Ge et al. 2020; Carey et al. 2024) and can significantly inhibit the proliferation, migration, and tube formation of human brain microvascular endothelial cells, which is significantly related to poor postoperative angiogenesis in adult MMD patients (He et al. 2022). Supplementation with creatine, while providing energy to cells within the body, may also restore the metabolic balance to a certain extent.

Phosphocreatine is the phosphorylated form of creatine, which serves as a key molecule in energy storage and transfer. Its blood level reflects the energy reserve status of muscles and other tissues (Tang et al. 2023). Phosphocreatine in the blood primarily exists in its free form rather than specifically in the form of disodium creatine phosphate. Therefore, the concentration of disodium creatine phosphate in blood is not directly used as an indicator of phosphocreatine concentration (Anders et al. 2021). This is consistent with our results, indicating that there is no direct linear relationship between creatine and disodium creatine phosphate.

The conversion of creatine and its phosphorylated derivatives into creatinine within the body is a spontaneous and irreversible process that occurs within muscle tissue (Wyss and Kaddurah‐Daouk 2000). Creatinine, a metabolic waste product, is transported to the kidneys via the bloodstream, where it is filtered from the blood and excreted in the urine. Its production rate is relatively fixed and is primarily determined by the total muscle mass and the rate at which creatine is converted to creatinine. Therefore, the concentration of creatinine in the blood is commonly used as an indicator of kidney filtration function (i.e., glomerular filtration rate [GFR]) and can indirectly reflect creatine metabolism and muscle mass (Rutherford et al. 2010). In our study, we observed that low creatine concentrations were associated with higher creatinine levels, while there was no significant difference in GFR between the groups with and without cerebrovascular events. This phenomenon may result from increased tissue utilization of creatine in patients, leading to greater creatinine production and, consequently, reduced blood creatine levels and elevated creatinine concentrations. In addition, the fact that the low creatine concentration group had a higher proportion of males may be related to this association. A low creatine concentration in patients with MMD may indicate cerebral vascular stenosis, which leads to ischemia and hypoxia, thereby increasing tissue energy utilization. For these patients, endogenous creatine production may not be sufficient to meet demand, and supplementation with exogenous creatine could be a new preoperative treatment strategy to improve ischemic and hypoxic conditions.

Supplementation with creatine can increase reserves in the brain (Forbes et al. 2022), suggesting that creatine supplementation is a potential therapeutic strategy for reducing the risk of adverse postoperative events in MMD. Although its effectiveness in the clinical treatment of MMD needs to be validated through cohort studies, the benefits of creatine supplementation have been confirmed in animal experiments and in the treatment of other diseases (Candow et al. 2023; Turner et al. 2015; Snow et al. 2020). Notably, the measurement of blood creatine levels in our study was conducted at 8:00 a.m. after a 12‐h fast to eliminate the influence of diet and vigorous exercise. This method accurately reflects the endogenous creatine concentrations in the body. Based on the data analysis in our study, the preoperative creatine concentration of patients should exceed the median level among all patients with MMD, preferably reaching the highest level of the third tertile to have a statistical difference in reducing the occurrence of postoperative cerebrovascular events.

In our study, we stratified participants into tertiles based on their serum creatinine levels and adjusted for variables pertinent to the prognosis of cerebrovascular events, including SBP, hyperlipidemia, RBC count, FDP, and total CO_2_. Using these adjusted variables, we constructed a nomogram to facilitate medical decision‐making by enabling the monitoring or modification of these prognostic indicators, potentially improving patient outcomes. However, the efficacy and applicability of this approach necessitate further validation through randomized controlled trials (RCTs), which remain the focus of our forthcoming research endeavors.

Although our study specifically focused on patients with MMD, it is pertinent to consider how postoperative outcomes differ across various cerebrovascular conditions treated with neurosurgical interventions. Diseases such as cerebral aneurysms and arteriovenous malformations (AVMs) present distinct pathophysiological mechanisms and surgical challenges compared with MMD. For instance, aneurysm surgeries often involve clipping or endovascular coiling to prevent rupture, whereas AVM treatments may require embolization or resection to eliminate abnormal vascular connections (Brisman et al. 2006; Lawton et al. 2015). These differences can influence postoperative recovery, complication rates, and long‐term outcomes. Existing literature suggests that the risk factors for postoperative events can vary significantly between these conditions. For example, aneurysm size and location may play a more critical role in aneurysm surgery than in MMD interventions (Tawk et al. 2021). Incorporating serum creatine levels as prognostic markers in these diverse contexts could provide insights into whether the predictive value of creatine is specific to MMD or extends to other cerebrovascular neurosurgeries. We aim to conduct comparative studies in the future to clarify these potential differences and improve the generalizability of creatine as a biomarker across various cerebrovascular conditions.

While our study demonstrated a significant association, it did not establish a causal relationship between serum creatine levels and cerebrovascular event risk. Several factors, such as residual confounding, measurement variability, and the observational nature of the study, limit our ability to infer causality. Prospective cohort studies and RCTs investigating creatine supplementation in patients with MMD are warranted to clarify the causal role of creatine in the risk of cerebrovascular events. Such studies could provide more definitive evidence regarding the therapeutic potential of creatine in reducing postoperative cerebrovascular events.

## 5 Conclusion

This study investigated the correlation between serum creatine concentrations and the incidence of adverse cerebrovascular events in patients with MMD following surgical intervention. These findings suggest a significant association between lower serum creatine levels and an elevated risk of cerebrovascular events. Consequently, creatine supplementation may emerge as a promising therapeutic approach for mitigating the risk of postoperative cerebrovascular events in patients diagnosed with MMD.

## Author Contributions

**Siqi Mou**: conceptualization, investigation, formal analysis, writing–original draft. **Zhikang Zhao**: methodology, writing–review and editing. **Chenglong Liu**: methodology, writing–review and editing. **Junsheng Li**: data curation. **Qiheng He**: data curation. **Wei Liu**: data curation. **Bojian Zhang**: data curation. **Zhiyao Zheng**: data curation. **Wei Sun**: data curation. **Xiangjun Shi**: data curation. **Qian Zhang**: resources. **Rong Wang**: resources. **Yan Zhang**: resources. **Peicong Ge**: project administration. **Dong Zhang**: project administration, funding acquisition.

## Ethics Statement

This study was conducted in accordance with the Declaration of Helsinki and approved by the Ethics Committee of Beijing Tiantan Hospital.

## Consent

Informed consent was obtained from all subjects involved in the study.

## Conflicts of Interest

The authors declare no conflicts of interest.

### Peer Review

The peer review history for this article is available at https://publons.com/publon/10.1002/brb3.70331

## Data Availability

The datasets used and/or analyzed in the current study are available from the corresponding author upon reasonable request.
